# Evaluating the use of BodyWorks Eve® high-fidelity ultrasound simulation equipment in formative clinical assessments

**DOI:** 10.1177/1742271X251320549

**Published:** 2025-03-12

**Authors:** Jane Arezina, Sandra Morrissey, Wendy Harrison

**Affiliations:** 1 Faculty of Medicine and Health, University of Leeds, Leeds, UK; 2 Leeds Institute of Medical Education, Leeds, UK; 3 Specialist Science Education Department, Leeds Institute of Cardiovascular and Metabolic Medicine, Leeds, UK

**Keywords:** Simulation, ultrasound, clinical, learning, education

## Abstract

**Introduction:**

Increasing demand for ultrasound services is reducing learners’ access to medical ultrasound clinical experience. High-fidelity simulation equipment, such as the BodyWorks Eve®, enhances the learners’ experience and scanning ability. This has the potential to improve patient safety as the learners’ ability to detect, identify and accurately report a known pathology can be assessed, which is not possible in clinical practice.

**Methods:**

Participants performed one pathological ultrasound examination on the BodyWorks Eve® and the participants’ performance level was assessed by the primary investigator using a formative clinical assessment form already used by the Diagnostic Imaging programme at the University of Leeds. The outcome was analysed using narrative statistics, and participants’ feedback was evaluated using thematic analysis.

**Results:**

A total of 16 participants were recruited. Eight (50%) reached the required level, but eight (50%) failed to reach the required level in at least one of the seven criteria that indicate professionally incompetent or dangerous practice. Thematic analysis of all the participants’ comments identified four main themes and two sub-themes which highlighted the benefits of the simulated assessment for prompting reflection, replicating clinical practice and gaining confidence in the assessment process, while also identifying negative aspects such as technical limitations when using the BodyWorks Eve®.

**Conclusion:**

Most participants evaluated the BodyWorks Eve® favourably. Using BodyWorks Eve® for formative clinical assessments is feasible and acceptable to participants. Further correlation to outcomes in clinical practice would be useful.

## Introduction

Sonographers use cutting-edge medical imaging technology to undertake, interpret and report on a wide range of complex medical ultrasound examination.^1^ Sonography is constantly evolving and significant advances over the last 25 years suggest it could become a profession in its own right.^1^

The number of clinical placements offered by ultrasound service providers is limited, partially due to the high demand for medical ultrasound examinations.^2^ Increasing demand, waiting lists and staff vacancies cause significant pressures on clinical ultrasound services and will potentially continue to restrict ultrasound training programme numbers as many departments see training of learners as an additional burden.^3^ In 2019, ultrasound was the second most common imaging test in the United Kingdom with 0.81 million ultrasound examinations performed.^3^ The number of non-obstetric ultrasound examinations increased by 10.3% between 2022 and 2023 and those waiting more than 6 weeks increasing by 2.2% from 18.6% to 20.9%.^3^ This is despite a national shortage of qualified sonographers^2^ and reported vacancy rates of up to 14.9%.^4^

Sonography education in the United Kingdom is predominantly at postgraduate level. Most trainees are from healthcare backgrounds with equally high vacancy rates such as diagnostic radiography and midwifery,^4^ which threatens to undermine the effectiveness of the clinical placement experience.^5^

Simulation is used widely in health care education, which is integral to the delivery of many medical ultrasound programmes. It provides more diverse learning opportunities and is a recognised innovative pedagogic approach to learning.^6^ Ultrasound simulation includes phantoms that reproduce body parts (with or without pathology) and more complex high-fidelity ultrasound imaging simulators such as BodyWorks Eve® (BWE).^7,8^ BWE is an ultra-realistic female patient simulator with palpable, accurate anatomical landmarks and over 100 real patient cases including 10,000 pathology variations, which allows learners to perform realistic ultrasound examinations. The inclusion of simulation in the curriculum ensures that a variety of teaching approaches are utilised, which increases inclusivity for learners.^9^ Simulation standardises learning and has the potential to improve safety as it enables learners to make mistakes without putting patients at risk. In addition, it promotes critical thinking and provides opportunities for feedback.

Studies show that a combination of simulation alongside supervised clinical practice produces positive learning outcomes,^10^ increases learners’ confidence^11^ and competence^7,10^ and is twice as effective as traditional supervised clinical experience as it provides a more concentrated learning environment.^12^ The postgraduate Diagnostic Imaging (DI) programme at the University of Leeds utilises simulation to give learners the opportunity to engage in enterprising, innovative and creative activities while augmenting clinical practice experience thereby reducing the burden on ultrasound service providers.

Simulation provides a realistic training environment, which helps learners to integrate theoretical knowledge with clinical experience.^13^ It has been proposed as a valid and reliable method for assessment of clinical ultrasound skills which compliments assessments undertaken in the clinical placement.^14^ As early as 2008, Lammers et al.^15^ identified that simulation allowed educators to develop learner-focused training and outcomes-based assessments. Weller et al.^16^ predicted that advances in simulation technology would increase its ability to replicate real clinical experience and potentially help to resolve capacity issues in clinical education.^17^

Clinical assessments are usually undertaken in the clinical environment on ‘real’ patients, and mandatory assessment of competency to practice via a summative clinical assessment prior to qualification is an essential component of any medical ultrasound programme accredited by the Consortium for the Accreditation of Sonographic Education (CASE).^18^ Formative (or ‘mock’) clinical assessments which replicate the summative assessments are undertaken to monitor progress, to enable learners to reflect on their performance and to provide a format for structured feedback.

CASE acknowledges that simulation has a role in formative learning but should not replace the assessment of clinical skills in the clinical setting.^18^

The purpose of this study was to investigate whether it was feasible for participants to undertake a formative clinical assessment on the BWE that could replicate undertaking an assessment in clinical practice. The study also aimed to undertake thematic analysis of participants’ feedback.

## Method

Formative clinical assessments were conducted by the primary investigator (PI), who was an experienced sonographer and medical ultrasound lecturer who had been undertaking clinical assessments for 20 years. The data were analysed using narrative statistics, and participants’ feedback was evaluated using thematic analysis.^19^

Purposive sampling was utilised to recruit 16 participants (9 females and 7 males)^20^ from a cohort of the 28 learners all of whom were enrolled on the DI programme and undertaking at least one of the following clinical modules: Obstetric, Gynaecological or General Medical Ultrasound.^21^ A total of 14 participants were diagnostic radiographers; 2 were medical doctors all of whom had been scanning for a minimum of 14 hours per week for a minimum of 3 months.

Ethical approval was gained prior to commencing the study (MREC number: 19-011; 7 January 2020). All the students in the cohort were sent the participant information, and consent to use all information gained during the research was obtained from the 16 participants via the participant consent form. Participants’ rights to confidentiality, safeguarding, data protection and withdrawal were discussed prior to commencing the study and an opportunity to ask questions was provided. All participants had a debrief discussion with the PI and information which included organisations and sources of support was provided.

Participants were randomly allocated a pathological case linked to one of the clinical modules being undertaken by each participant; three participants were allocated an obstetric case: four a gynaecological case and nine a general medical case. All 16 formative clinical assessments were conducted over a period of 5 days by the PI who assessed and recorded the participants’ performance against pre-determined criteria using the performance level descriptors (Table 1) used by the DI programme (see Supplemental Appendix 1). Seven criteria that indicate professionally incompetent or dangerous practice were identified on the assessment form by double-stars (**). If participants failed to achieve a level 2 or above in any of these seven criteria, this would result in a fail had this been a summative assessment. Participants reflected on their performance and provided written comments regarding their experience of undertaking the formative assessment immediately after the assessment and before the outcome of the assessment were shared. The PI then informed the participants of the outcome of the assessment and provided verbal and written feedback to the participants. This was followed by a structured debrief discussion, which enabled participants to reflect on the assessment experience, to identify lessons learned and to formulate a plan for improvement.

**Table 1.** Performance-level descriptors.

1. Works below the expected level Limited ability to fulfil the performance criteria, the participant requires frequent direct supervision and advice to perform the examination. 2. Works at the expected level The performance fulfils the performance criteria, there is evidence of integration of knowledge and skill required to perform competently without supervision or requires minimal advice. 3. Works above the expected level The performance fulfils the performance criteria, the participant integrates conceptual knowledge and skills, plus there is evidence of critical appraisal and reflective abilities without supervision. X-Not performed

Included with the permission of University of Leeds.

All completed formative assessment forms were anonymised. Narrative statistics were reported along with a comparison of the number and proportions of fails across the different clinical areas. A Fisher’s exact test^22^ was performed to assess whether these proportions were statistically significant. Thematic analysis was applied to the full dataset^19,23^ and the PI analysed and openly coded the transcripts. Codes were assigned according to their characteristics and formalised into the main themes. Concepts were then categorised and ordered into themes and sub-themes that reflected the research aims and questions. Representative quotes were selected for each theme and sub-theme.^24^

## Results

### Outcome of the formative clinical assessment

Eight of the 16 (50%) participants recruited would have passed if this had been a summative clinical assessment. However, 50% (eight participants) failed to reach the required level for at least one of the areas denoted by double-stars and would have failed (Table 2).

**Table 2.** Participants who failed to reach the required level in areas that indicate professionally incompetent or dangerous practice.

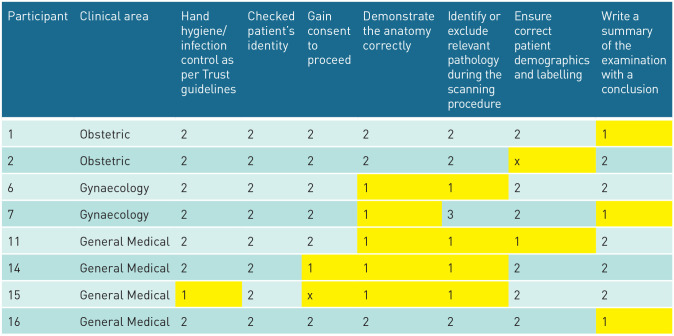

Included with the permission of the University of Leeds. Key

1. Works below the expected level.

2. Works at the expected level.

3. Works above the expected level.

X. Not performed.

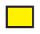
indicates automatic fail at summative assessment.

Of these, two (25%) examined an obstetric case, two (25%) a gynaecological case and four (50%) a general medical case. Comparison of fails across the different clinical areas identified that of the three obstetric cases, two participants failed (66.7%); of the four gynaecological cases, two participants failed (50.0%); and of the nine general medicine cases, four participants failed (44.4%). There was no statistically significant difference in the proportion of fails across clinical areas (Fisher’s exact *p* ⩽ 1.000) and no benefit to performing additional analysis, given the large *p*-value.^23^

Three participants did not achieve the required level for one criterion (P1, P2 and P16). Two participants did not achieve the required level in two criteria (P6 and P7).

Two participants did not achieve the required level in three criteria (P11 and P14). One (participant 15) did not achieve the required level in four criteria.

### Thematic analysis of the feedback from all of the participants

**Figure 1.**
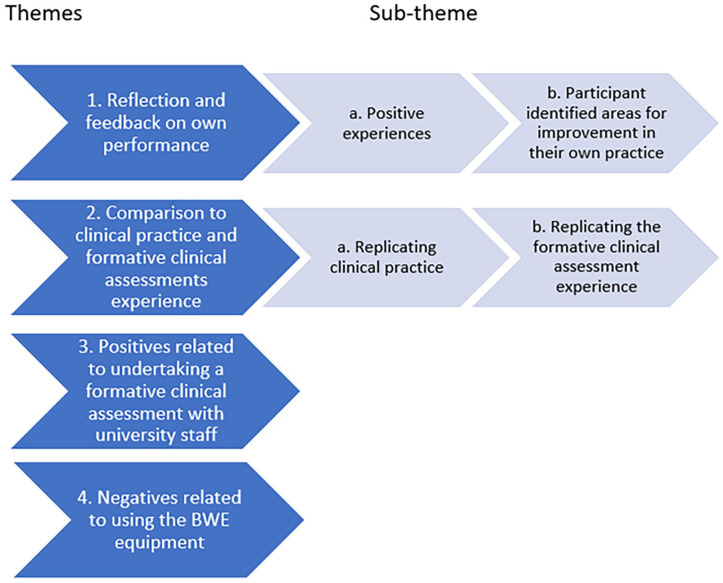
Themes and sub-themes.

Four main themes and two sub-themes emerged from the thematic analysis of the participants’ feedback (see Figure 1).

Representative quotes have been included from all the participants, regardless of whether they reached the required level or not. Where the quote is from a participant who failed to the required level in one or more criteria, the letter ‘F’ has been added after the quote.

#### Participant feedback on their performance

##### Positive experiences

Most participants stated that they had gained something positive from using the simulation for a formative clinical assessment. One participant (P13) stated ‘I enjoyed the whole experience’ and another stated that ‘The assessment is good’ (P5).

Some participants were confident that they had performed or communicated well during the assessment. One participant stated that ‘I believe my scan went well’ (P10) and another that ‘Overall it is a good formative’ (P5). Other comments included:

‘Spoke to the patient well and explained the pathology adequately.’ (P3)

‘My professionalism was good. Asked appropriate questions.’ (P9)

‘My communication I felt was good as I talk to the patient via procedure.’ (P14 (F))

##### Participant identified areas for improvement in their own practice

Most participants reflected on their performance to identify areas for improvement. One participant stated, ‘Need more practice scanning’ (P12 (F)).

Many participants identified specific areas that needed improvement:

‘. . . felt like it has pointed where I need to focus my time on e.g. studying their anatomy.’ (P15 (F))

‘I think that the examination went well, however, there is still room for improvement. Thinking about landmarks will help me with some aspects.’ (P11 (F))

‘My overall confidence with doing abdominal scans is still developing as general medical US is the area I have the least experience in. I still find the liver in particular quite difficult as it is such a large organ with lots of structures to assess.’ (P13)

‘Overall I was very nervous, I need in future to get and focus on my landmarks.’ (P14 (F))

One participant stated that it had given them the opportunity to scan pathology that they had not seen in clinical practice:

‘. . . it gave me a chance to assess anatomy that I may not see during my training. I have learnt how to fully examine that anomaly and I would feel more confident if I was to come across it in practice.’ (P1 (F))

Other participants mentioned issues relating to image optimisation or use of equipment features that may enhance the examination:

‘The session has highlighted to me the importance of using my depth correctly and using sector width.’ (P4)

‘Remember to use the colour Doppler. Knowledge of malignant ovarian masses. Persevere on finding ovaries on TA.’ (P6 (F))

‘Difficult assessment of the kidneys, I need to work on image optimisation and using functions such as colour Doppler and measurements and disabling compound imaging.’ (P9)

‘I could have done better in terms of image optimisation and assessment of uterus and ovaries.’ (P10)

Other participants identified areas of examination technique that had affected their performance:

‘Future-don’t panic and also key thing is look at the scan in a broader view, so I don’t miss pathology on the periphery.’ (P14 (F))

‘Not to be nervous and more decisive at point of time with knowing when to move on and not fixate on a particular structure. Also measure pathology and ensure examiner is aware that pathology has been noted. Improve image quality -better sections of organs e.g. kidneys TS and aorta.’ (P8)

Some participants felt that the feedback would reduce mistakes when performing the assessment in practice:

‘The feedback will be with me now and I know what to do/not to do in the exam. I made mistakes during simulation which I now know how to correct in the real exam. Useful to see the images and discuss how to improve them, I will definitely concentrate more on image optimisation.’ (P2 (F))

#### Replicating clinical practice and formative clinical assessments experience

##### Replicating clinical practice

Overall, most participants felt that scanning the BWE was similar to undertaking an examination in clinical practice:

‘The scanning is true to real life.’ (P1 (F))

‘Overall, a work-like learning experience.’ (P7 (F))

One participant stated that

‘. . . I am pleased that I set the patient on the correct clinical pathway.’ (P10)

Most participants who comment on this aspect felt that the simulation had helped them to practice their skills in a safe environment and increased their confidence in specific areas:

‘BodyWorks Eve was helpful to practice clinical skills in a no-pressure environment.’ (P4)

‘Todays [sic] session has given me confidence in the hospital setting in understanding protocols and utilising US to diagnose pathology.’ (P4)

##### Replicating the formative clinical assessment experience

Many of the participants felt that using the BWE was a useful adjunct, not an alternative, to performing formative assessments on ‘real’ patients in a clinical setting:

‘I think that BodyWorks would work well alongside real formative examinations.’ (P7 (F))

‘Very useful to experience simulated exam scenario.’ (P2 (F))

‘Exercise is a good indicator of assessment expectations and ‘stress’.’ (P16 (F))

‘Useful to take the exam under exam conditions. Made me think on the spot and great practice for the real thing.’ (P12)

‘I liked how we could discuss what we should and shouldn’t do/say during an assessment.’ (P7 (F))

#### Opportunity to do a formative assessment with university staff

Some participants liked the opportunity to show their skills and receive feedback from a member of university staff:

‘This session was useful as it gave the PI a chance to see how my scanning ability is at this stage and she offered some useful advice.’ (P13)

‘I didn’t feel under pressure and felt comments made by the PI were supportive and helpful.’ (P15 (F))

‘Helps to chat through anatomy and technique. Would like to do more throughout the year.’ (P16 (F))

#### Negatives identified with the equipment

Some participants identified areas that might improve the authenticity of the BWE such as the inclusion of transvaginal (TV) capability and a variety of technical improvements:

‘Could be enhanced with a TV scan to confirm diagnostic accuracy.’ (P3)

In addition, many of the participants felt that scanning the BWE was not the same as scanning real patients, making it difficult to demonstrate their skills in this setting as opposed to in the clinical setting:

‘. . . as BodyWorks Eve is immobile and the technical controls are very different to an US machine. It was difficult to portray what I can do in clinical practice with BodyWorks Eve.’ (P13)

‘. . . found it more difficult to scan dummy so become more adapted to it.’ (P8)

‘Tendency to forget things in this scenario since the patient is not real and less adaptions.’ (P5)

## Discussion

Evidence suggests that simulation is a good adjunct to clinical experience,^6,7,9,11–17,25^ but there is limited research regarding the assessment of clinical skills development of medical ultrasound students.^26^

Simulation creates a safe training environment^13^ that closely replicates clinical practice^27^ and allows learners to acquire the skills required for practice^28^ and to actively participate in their own learning.^13^ Most participants felt that performing a formative clinical assessment using BWE enabled them to practice their skills in a safe, supportive environment.^29^

Eight participants (50%) were assessed as competent to practice while the other 50% would have failed in areas classed as professionally incompetent or dangerous practice. However, the purpose of formative assessments is not to confer competence but to evaluate the level of learning achieved for a specific skill, whereas summative assessment assesses whether that skill has been mastered.^30^ At the time of the study, the participants had been scanning for a short period of time and one participant (P12) stated that they had not had sufficient scanning experience. For participant 13, this was linked to a specific clinical area. The participants’ level of experience in each clinical area varied; however, the proportion of fails in the different areas was not found to be statistically significant (*p* ⩽ 1.000),^23^ probably due to the small number of participants.

Simulation provides an authentic hands-on experience that requires students to apply their knowledge and skills to real-world situations and authenticity is not only crucial to the development of decision-making, clinical judgement and clinical reasoning skills^31^ but also enhances the learners’ ability to cooperate with the interdisciplinary team, to manage complex situations and to understand interpersonal relations.^13^ Self-confidence, which is the ability to demonstrate essential clinical practice skills, also increases learners’ satisfaction, self-efficacy and capacity for critical thought^31^ and two participants were confident that they had performed well (P5, P10).

A structured debrief discussion is an essential component of the formative assessment process,^13^ as it allows learners to reflect on their performance which is essential for learning to occur.^32,33^ Some participants were able to reflect on their performance and self-identify areas for development^34^ while others (P13, P15 and P16) valued receiving feedback from university staff. It enables them to identify areas for improvement lessons and to formulate an action plan.

Most participants received the feedback positively, which is in line with other studies, where learners expressed positive sentiments about undertaking formative assessments and receiving feedback.^35^ Positive feedback and encouragement can boost learners’ confidence; however, for negative feedback to be perceived constructively, the simulation environment needs to foster an atmosphere of mutual respect and cooperation.^31^

BWE was perceived as a positive addition, but not an alternative, to the formative assessment undertaken on patients in a clinical setting.^28^ The evidence from this study suggests that simulation could replace some aspects of the clinical training and formative clinical assessments and is likely to be welcomed by learners and ultrasound departments alike^2^ as it will help learners prepare for the assessment and may potentially reduce pre-assessment anxiety.

Communication is integral to the quality of care and simulation can help to develop these skills.^13^ Evidence shows that communication skills development helps learners to interact effectively with patients and positively influences the quality of the healthcare they provide.^31^ Participants articulated their feelings and perceptions relating to communication and several of the participants (P3, P9 and P14) expressed confidence in their ability to communicate well with the ‘patient’, despite this being a manikin. Sonographers are often the first health professional to identify and deliver news of pregnancy complications to parents.^36^ Communication coaching could support sonographers when delivering unexpected news^37^ and a recent independent review into pregnancy loss^38^ recommended that all sonographers should undertake training to develop their communication skills. Research exploring the use of BWE communication coaching with learners to enhance their communication skills is recommended.

The more realistic simulation is, the greater the learning.^17,39^ Aspects of the BWE made the assessment less authentic, such as differences in the system controls compared to ultrasound equipment and not having the capability for TV examinations.

Aligning the BWE to scanning in clinical practice is important as BWE could be potentially extremely useful as an adjunct to clinical placement experience,^40^ in formative clinical assessments and as a major resource for ultrasound training programmes.

## Limitations of the study

Low participant numbers (*n* = 16) may have reduced the statistical power of the study. It would be useful to undertake further research with a higher number of participants.

Using only one assessor was a limitation of this study as increasing the number of assessors improves the accuracy and reliability of the assessment.^40^ In practice, learners undertaking clinical assessments have two assessors and are assessed on three cases for each clinical module. In this study, participants were only assessed on one clinical case and the short duration of the assessment may have negatively impacted on its usefulness.^39^

The study planned to compare the participants’ performance on BWE to the outcomes of the formative clinical assessments undertaken in clinical practice to ascertain whether the participants used the feedback and feed forward gained during the study.^33^ However, this was not possible due to the Covid-19 pandemic.

## Implications for research and practice

A study replicating the formative clinical assessment undertaken in clinical practice, including the same number of cases and using two assessors, is recommended to improve reliability and could also explore whether participants used the assessors’ simulation feedback or feed forward when undertaking formative clinical assessments in clinical practice.^13^ It may be useful to explore whether simulation could be used for summative clinical assessments, while taking the importance and implications of conferring competence to practice into account.^27^

Although not related to assessment, a study to investigate the use of BWE to facilitate communication coaching^38^ would be pertinent as communication is an extremely important aspect of a sonographers’ role in line with a recent independent review into pregnancy loss.^38^

## Conclusion

This study indicates that using simulation to perform formative clinical assessments is feasible and was positively received by the participants. Extending the study to include more cases, additional assessors and comparing participants’ performance during simulation to their performance in clinical practice would increase the rigour of a future study. Further studies into whether it is appropriate to use simulation for summative clinical assessments and for communication coaching are also recommended.

## Supplemental Material

sj-docx-1-ult-10.1177_1742271X251320549 – Supplemental material for Evaluating the use of BodyWorks Eve® high-fidelity ultrasound simulation equipment in formative clinical assessments Supplemental material, sj-docx-1-ult-10.1177_1742271X251320549 for Evaluating the use of BodyWorks Eve® high-fidelity ultrasound simulation equipment in formative clinical assessments by Jane Arezina, Sandra Morrissey and Wendy Harrison in Ultrasound
