# Supplementary material for: Evaluating the use of BodyWorks Eve® high-fidelity ultrasound simulation equipment in formative clinical assessments
Source: Ultrasound. 2025 Mar 12;33(4):301–9. doi: 10.1177/1742271X251320549 (PMC11907560; doi:10.1177/1742271X251320549)
Supplement: sj-docx-1-ult-10.1177_1742271X251320549 – Supplemental material for Evaluating the use of BodyWorks Eve® high-fidelity ultrasound simulation equipment in formative clinical assessments [file sj-docx-1-ult-10.1177_1742271X251320549.docx]

**Supplementary material 1**: **Postgraduate Certificate/ Postgraduate Diploma/ MSc Diagnostic Imaging Formative Clinical Assessment form. Included with the permission of the University of Leeds**

| **Postgraduate Certificate/ Postgraduate Diploma/ MSc Diagnostic Imaging** | **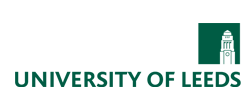** |
| --- | --- |

**Formative Clinical Assessment**

**Clinical Ultrasound modules**

| **Module** |
| --- |
| **Student Name** |
| **Placement Site** |
| **Date** |
| **Internal examiners** |
|  |
|  |

Please note the following:

- This is a formal examination and will follow examination procedures according to the University of Leeds Assessment Guidelines
- There is an obligation to bring any matters affecting performance to the attention of the Student Education Support Office in writing prior to the examination commencing. There is no obligation to communicate these matters to the internal examiners
- Be aware of the items that may indicate professionally incompetent or dangerous practice. These are denoted by **
- If you have any questions, please ask these prior to the start of the examination.
- At least ONE form to be signed by the internal examiners and the student at the end of the assessment and uploaded to PebblePad
- After the final formative clinical assessment, both internal examiners and the student to complete the declaration of agreement to proceed to the summative clinical assessment on PebblePad.

Per assessment:-

- **Three** patients are compulsory for each module with three different reasons for referral

1. **This form should be completed by the supervising mentor in order to evaluate the safe and proficient practice of students whilst undertaking a range of ultrasound examinations.**
2. **Three patients are compulsory for each module.**
3. **Three different reasons for referral are required.**

**4a. The form should be completed at the mid-point of the module to check progress and to identify action points for the student’s practice. The student should be working towards the expected levels of performance.**

**4. b. The form should also be completed 2-4 weeks prior to the summative clinical assessment. This will inform the decision to proceed to summative clinical assessment. Complete the ‘Declaration of agreement to proceed to summative clinical assessment’**

1. **Where the student is working ‘below the expected level’ the module leader, student and mentor will need to discuss the required action.**
2. **The** **Declaration of Agreement to proceed must be** **uploaded to PebblePad prior to the summative assessment.**

**Expected levels of performance**

For the formative assessments, the levels are defined as;

**1 Works below the expected level**

Limited ability to fulfil the performance criteria, the student requires frequent direct supervision and advice to perform the examination.

**2 Works at the expected level**

The performance fulfils the performance criteria, there is evidence of integration of knowledge and skill required to perform competently without supervision or requires minimal advice.

**3 Works above the expected level**

The performance fulfils the performance criteria, the student integrates conceptual knowledge and skills, plus there is evidence of critical appraisal and reflective abilities without supervision.

(** denotes areas that may indicate professionally **incompetent** or **dangerous** practice)

The student is assigned a performance level. **Circle the number relating to performance in each box for each patient**

The performance levels are a means to identify the student’s strengths and weaknesses. The formative assessment may be used by the student and clinical mentor to monitor and discuss progress, and then develop an action plan for the clinical learning.

**SECTION 1 IMAGING REQUEST**

This part of the Assessment is to be discussed with the student prior to the examination.

Can the student: -

|  | **Patient number** | **1** | **2** | **3** |
| --- | --- | --- | --- | --- |
| i | Explain why the request was made | 1/2/3 | 1/2/3 | 1/2/3 |
| ii | Understand the implications of the condition(s) being investigated | 1/2/3 | 1/2/3 | 1/2/3 |
| iii | Obtain all relevant information  With reference to the following:   - Importance of other sources for clinical history (findings) - Justification of the request - Evidence based practice | 1/2/3 | 1/2/3 | 1/2/3 |
| iv | Plan the examination appropriately based on local/professional guidelines   - Possible manipulation of equipment, room layout and adaptation of the technique | 1/2/3 | 1/2/3 | 1/2/3 |

**COMMENTS**

| **Patient 1** |
| --- |
| **Patient 2** |
| **Patient 3** |

**SECTION 2 THE ULTRASOUND EXAMINATION**

This section is to be observed and scored in silence as the student performs the examination.

**2a Before the examination**

Does the student:

|  | Start time |  |  |  |
| --- | --- | --- | --- | --- |
|  | Patient number | **1** | **2** | **3** |
| i | Introduce him/herself and the internal examiners | 1/2/3 | 1/2/3 | 1/2/3 |
| ii | Hand hygiene/infection control as per Trust guidelines** | 1/2/3 | 1/2/3 | 1/2/3 |
| iii | Check the patient’s identity** | 1/2/3 | 1/2/3 | 1/2/3 |
| iv | Obtain a verbal clinical history | 1/2/3 | 1/2/3 | 1/2/3 |
| v | Explain the procedure to the patient; Give clear, precise instructions to the patient | 1/2/3 | 1/2/3 | 1/2/3 |
| vi | Provide opportunity for questions from the patient/companion throughout the examination | 1/2/3 | 1/2/3 | 1/2/3 |
| vii | Gain consent to proceed** | 1/2/3 | 1/2/3 | 1/2/3 |
| viii | Check on the required preparation for this examination | 1/2/3 | 1/2/3 | 1/2/3 |
| ix | Communicate with the patient in a professional and sensitive manner, including appropriate care | 1/2/3 | 1/2/3 | 1/2/3 |
| x | Treat the patient appropriately with respect to privacy and dignity | 1/2/3 | 1/2/3 | 1/2/3 |

**2b. During the examination**

Does the student

|  | Patient number | **1** | **2** | **3** |
| --- | --- | --- | --- | --- |
| i | Set up the equipment and room initially e.g. presets; patient demographics; infection control; ergonomics | 1/2/3 | 1/2/3 | 1/2/3 |
| ii | Use a systematic approach and select the appropriate technique(s) to perform a high quality/diagnostic examination | 1/2/3 | 1/2/3 | 1/2/3 |
| iii | Demonstrate competence in the use and adaptation of the equipment during the examination | 1/2/3 | 1/2/3 | 1/2/3 |
| iv | Demonstrate the anatomy correctly** | 1/2/3 | 1/2/3 | 1/2/3 |
| v | Identify or exclude relevant pathology during the scanning procedure** | 1/2/3 | 1/2/3 | 1/2/3 |
| vi | Adapt the technique as required by their observations/verbal patient history e.g. additional patient positions/manoeuvres | 1/2/3 | 1/2/3 | 1/2/3 |
| vii | Communicate with the patient in a professional and sensitive manner, including appropriate care | 1/2/3 | 1/2/3 | 1/2/3 |
| viii | Record relevant good quality images at the appropriate points of the examination for the discussion in section 3 | 1/2/3 | 1/2/3 | 1/2/3 |
| ix | Manage the examination time appropriately | 1/2/3 | 1/2/3 | 1/2/3 |
| x | Review the images prior to the patient leaving the department   - Ensure correct patient demographics and labelling ** - Write a summary of the examination with a conclusion ** | 1/2/3 | 1/2/3 | 1/2/3 |
|  |  | 1/2/3 | 1/2/3 | 1/2/3 |

**2c. After the examination**

Does the student:

|  | Patient number | 1 | 2 | 3 |
| --- | --- | --- | --- | --- |
| i | Discuss the results with the patient/companion or inform them who will do so (as per local guidelines) | 1/2/3 | 1/2/3 | 1/2/3 |
| ii | Attend to the aftercare of the patient and ensures that continuing care/appropriate patient management is provided as necessary | 1/2/3 | 1/2/3 | 1/2/3 |
|  | Finish time |  |  |  |

**SECTION 2 and 3** **COMMENTS**

| **Patient 1** |
| --- |
| **Patient 2** |
| **Patient 3** |

**Section 3**

This part of the assessment is to be discussed with the student on completion of the examination.

**Evaluations and Reflection**

Does the student:

|  | Patient number | 1 | 2 | 3 |
| --- | --- | --- | --- | --- |
| i | Write a final report | 1/2/3 | 1/2/3 | 1/2/3 |
| ii | Critically evaluate the examination with reference to:-   - Anatomy - Equipment - Image quality - Pathology - Safety - Technique | 1/2/3 | 1/2/3 | 1/2/3 |
| iii | Critically evaluate the usefulness/role of the ultrasound examination in relation to clinical findings, other imaging and patient management | 1/2/3 | 1/2/3 | 1/2/3 |
| iv | Reflect upon the overall examination quality | 1/2/3 | 1/2/3 | 1/2/3 |

**Student’s comments:**

|  |
| --- |

**Mentor’s comments:**

|  |
| --- |

**Action Plan**

|  |
| --- |

**Mentor’s signature: Date**

**Student’s signature: Date**

**Supplementary material 2** Outcome **of the formative clinical assessment**

| **P** | **Clinical area** | **1i** | **1ii** | **1iii** | **1iv** | **2ai** | **2aii**** | **2a iii**  ****** | **2a iv** | **2a v** | **2a vi** | **2a vii **** | **2a viii** | **2a ix** | **2a x** | **2b i** | **2b ii** | **2b iii** | **2b iv **** | **2bv **** | **2b vi** | **2b vii** | **2b viii** | **2b ix** | **2bx **** | **2bx **** | **2c i** | **2c ii** | **3 i** | **3 ii** | **3 iii** | **3iv** |
| --- | --- | --- | --- | --- | --- | --- | --- | --- | --- | --- | --- | --- | --- | --- | --- | --- | --- | --- | --- | --- | --- | --- | --- | --- | --- | --- | --- | --- | --- | --- | --- | --- |
| 1 | O | 2 | 2 | 2 | 2 | 2 | 2 | 2 | 2 | 2 | x | 2 | x | 2 | 2 | 2 | 2 | 1 | 2 | 2 | 1 | 2 | 2 | 2 | 2 | 1 | 2 | 1 | 1 | 2 | 2 | 2 |
| 2 | O | 2 | 2 | 2 | 2 | 2 | 2 | 2 | 2 | 3 | x | 2 | x | 2 | 2 | 2 | 2 | 2 | 2 | 2 | 1 | 2 | 2 | 2 | x | 2 | 2 | 2 | 3 | 2 | 2 | 2 |
| 3 | O | 2 | 2 | 2 | 2 | 2 | 2 | 2 | 2 | 2 | 2 | 2 | 2 | 2 | 2 | 2 | 2 | 2 | 2 | 2 | 2 | 2 | 1 | 3 | 2 | 2 | 3 | 2 | 3 | 2 | 2 | 2 |
| 4 | G | 2 | 2 | 2 | 2 | 2 | 2 | 2 | 2 | 2 | 2 | 2 | 2 | 2 | 2 | 2 | 2 | 2 | 2 | 2 | 2 | 2 | 2 | 2 | 2 | 2 | 2 | 2 | 2 | 2 | 2 | 2 |
| 5 | G | 2 | 2 | 2 | 2 | 2 | 2 | 2 | 2 | x | x | 2 | x | 2 | 2 | 2 | 2 | 2 | 2 | 2 | 2 | 2 | 2 | 2 | 2 | 2 | 1 | 2 | 2 | 2 | 2 | 2 |
| 6 | G | 2 | 2 | 2 | 1 | 2 | 2 | 2 | 2 | 1 | x | 2 | x | 2 | 2 | 2 | 2 | 1 | 1 | 1 | 1 | 2 | 2 | 2 | 2 | 2 | 1 | 2 | 1 | 2 | 2 | 2 |
| 7 | G | 2 | 2 | 2 | 1 | 2 | 2 | 2 | 2 | 2 | x | 2 | x | 2 | 2 | 2 | 2 | 2 | 1 | 3 | 2 | 2 | 2 | 2 | 2 | 1 | 2 | 2 | 1 | 2 | 2 | 2 |
| 8 | GM | 2 | 2 | 2 | 2 | 2 | 2 | 2 | 2 | 1 | 1 | 2 | x | 2 | 2 | 2 | 1 | 2 | 2 | 2 | 2 | 2 | 1 | 1 | 2 | 2 | 2 | 2 | 2 | 2 | 2 | 2 |
| 9 | GM | 2 | 2 | 2 | 2 | 2 | 2 | 3 | 3 | 2 | x | 2 | 2 | 2 | 2 | 2 | 2 | 2 | 2 | 3 | 2 | 2 | 2 | 3 | 2 | 2 | 2 | 2 | 2 | 2 | 2 | 2 |
| 10 | GM | 2 | 2 | 2/3 | 2/3 | 2 | 2 | 2 | 2 | 2 | 2 | 2 | x | 2 | 3 | 2 | 2 | 3 | 2 | 3 | 2 | 2 | 2 | 2 | 2 | 2 | 2 | 2 | 2 | 2 | 2 | 2 |
| 11 | GM | 2 | 2 | 2 | 2 | 2 | 2 | 2 | 2 | 2 | 2 | 2 | x | 2 | 2 | 2 | 1 | 2 | 1 | 1 | 2 | 2 | 2 | 2 | 1 | 2 | 2 | 2 | 2 | 2 | 2 | 2 |
| 12 | GM | 2 | 2 | 2 | 2 | 2 | 2 | 2 | 2 | 2 | x | 2 | x | 2 | 2 | 2 | 3 | 2 | 2 | 3 | 2 | 2 | 2 | 2 | 2 | 3 | 2 | 2 | 2 | 2 | 2 | 2 |
| 13 | GM | 2 | 2 | 2 | 2 | 2 | 2 | 2 | 2 | 2 | 2 | 2 | x | 2 | 2 | 2 | 2 | 2 | 2 | 3 | 2 | 2 | 2 | 2 | 2 | 2 | 2 | 2 | 2 | 2 | 2 | 2 |
| 14 | GM | 2 | 2 | 2 | 2 | 2 | 2 | 2 | 1 | 1 | 2 | 1 | x | 2 | 2 | 2 | 1 | 2 | 1 | 1 | 2 | 2 | 1 | 1 | 2 | 2 | 2 | 1 | 1 | 2 | 2 | 2 |
| 15 | GM | 2 | 2 | 2 | 2 | 2 | 1 | 2 | 2 | 1 | 2 | x | x | 2 | 2 | 2 | 1 | 1 | 1 | 1 | 1 | 1 | 2 | 2 | 2 | 2 | 2 | 2 | 2 | 2 | 2 | 2 |
| 16 | GM | 2 | 2 | 2 | 2 | x | 2 | 2 | 2 | 1 | 2 | 2 | 2 | 2 | 2 | 2 | 1 | 2 | 2 | 2 | 2 | 2 | 1 | 2 | 2 | 1 | 2 | 2 | 1 | 2 | 2 | 2 |

**Key:-**

P=Participant number

Clinical area: - O=Obstetric; G= Gynaecology; GM=General Medical

1. Works below the expected level

2. Works at the expected level

3. Works above the expected level

X. Not performed

** denotes areas that may indicate professionally incompetent or dangerous practice

Indicates automatic fail at summative assessment

**Supplementary material 3**

**Participant feedback on their performance**

**a. Positive experiences**

Table 3

| **Themes** | **Sub-themes** | **Key concepts** | **Participant Quotes** |
| --- | --- | --- | --- |
| **1.**  **Reflection and**  **feedback on own performance** | a. Positive experiences | • Positive experience, simulation worked well | ‘Spoke to the patient well and explained the pathology adequately’ [P3]  ‘My professionalism was good. Asked appropriate questions.’ [P9]  ‘My communication I felt was good as I talk to the patient via procedure.’ [P14]  ‘The assessment is good.’ [P5]  ‘Overall it is [sic] a good formative.’ [P5]  ‘I believe my scan went well.’ [P10] |

**b. Participant identified areas for improvement in their own practice**

Table 4

| **Themes** | **Sub-themes** | **Key concepts** | **Participant Quotes** |
| --- | --- | --- | --- |
| **1.**  **Reflection and**  **feedback on own performance** | b. Participant identified areas for improvement in their own practice | • Participants able to reflect on the assessment  • able to self-identify areas for improvement. | ‘Future-don’t panic and also key thing is look at the scan in a broader view so I don’t miss pathology on the periphery’ [P14]  ‘…felt like it has pointed where I need to focus my time on e.g. studying their anatomy.’ [P15]  ‘…it gave me a chance to assess anatomy that I may not see during my training. I have learnt how to fully examine that anomaly and I would feel more confident if I was to come across it in practice.’ [P1]  ‘The feedback will be with me now and I know what to do/not to do in the exam. I made mistakes during simulation which I now know how to correct in the real exam. Useful to see the images and discuss how to improve them, I will definitely concentrate more on image optimisation’. [P2]  ‘The session has highlighted to me the importance of using my depth correctly and using sector width.’[P4]  ‘Remember to use the colour Doppler. Knowledge of malignant ovarian masses. Persevere on finding ovaries on TA [P6]  ‘Difficult assessment of the kidneys, I need to work on image optimisation and using functions such as colour Doppler and measurements and disabling compound imaging.’ [P9]  ‘I think that the examination went well, however, there is still room for improvement. Thinking about landmarks will help me with some aspects. I am happy with the scanning of the spleen, kidneys, aorta and liver but some better representational images of the liver would be better’ [P11]  ‘My overall confidence with doing abdominal scans is still developing as general medical US is the area I have the least experience in. I still find the liver in particular quite difficult as it is such a large organ with lots of structures to assess’ [P13]  ‘Overall I was very nervous, I need in future to get and focus on my landmarks.’ [P14]  ‘I could have done better in terms of image optimisation and assessment of uterus and ovaries.’ [P10]  ‘This session I found very useful; it gave me a chance to assess anatomy that I may not see during my training. I have learnt how to fully examine that anomaly and I would feel more confident if I was to come across it in practice’ [P1]  ‘Not to be nervous and more decisive at point of time with knowing when to move on and not fixate on a particular structure. Also measure pathology and ensure examiner is aware that pathology has been noted. Improve image quality -better sections of organs e.g. kidneys TS and aorta’ [P8]  ‘…felt like it has pointed where I need to focus my time on e.g. studying their anatomy’ [P15] |

**Supplementary material 4**

**Replicating clinical practice and formative clinical assessments experience**

**a. Replicating clinical practice**

Table 5

| **Themes** | **Sub-themes** | **Key concepts** | **Participant Quotes** |
| --- | --- | --- | --- |
| **2.**  **Replicating**  **Clinical**  **practice**  **and**  **formative**  **clinical**  **assessments**  **experience** | a. Replicating clinical practice | • Experience similar to clinical practice  • Scanning similar to undertaking an examination in clinical practice  • Practice their skills in a safe environment and increased confidence | ‘The scanning is true to real life’ [P1]  ‘Overall a work-like learning experience.’ [P7]  ‘BodyWorks Eve was helpful to practice clinical skills in a no-pressure environment.’ [P4]  ‘Todays [sic] session has given me confidence in the hospital setting in understanding protocols and utilising USS to diagnose pathology’ [P4] |

**b. Replicating the formative clinical assessment experience**

Table 6

| **Themes** | **Sub-themes** | **Key concepts** | **Participant Quotes** |
| --- | --- | --- | --- |
|  | b. Replicating the formative clinical assessment experience | • Using the BodyWorks Eve was a useful addition to undertaking the formative assessment in clinical practice | ‘I think that BodyWorks would work well alongside real formative examinations’ [P7]  ‘Very useful to experience simulated exam scenario.’ [P2]  ‘Exercise is a good indicator of assessment expectations and ‘stress’.’ [P16]  ‘Useful to take the exam under exam conditions. Made me think on the spot and great practice for the real thing.’ [P12]  ‘I liked how we could discuss what we should and shouldn’t do/say during an assessment’ [P7] |

**Supplementary material 5**

**Opportunity to do a formative assessment with university staff**

Table 7

| **Themes** | **Sub-themes** | **Key concepts** | **Participant Quotes** |
| --- | --- | --- | --- |
| **3.Opportunity to do a formative assessment with university staff** |  | The simulation gave participants the opportunity to receive feedback from the lecturer from the university  • Opportunity to show the lecturer their skills  • Opportunity to receive feedback | ‘This session was useful as it gave Jane a chance to see how my scanning ability is at this stage and she offered some useful advice.’[P13]  ‘I didn’t feel under pressure and felt comments made by Jane were supportive and helpful.’ [P15]  ‘Helps to chat through anatomy and technique. Would like to do more throughout the year.’ [P16] |

**Supplementary material 6**

**Negatives identified with using the equipment**

Table 8

| **Themes** | **Sub-themes** | **Key concepts** | **Participant Quotes** |
| --- | --- | --- | --- |
| **4.Negatives identified with using the equipment** |  | Participants identified areas for improvement with the equipment  • Ways make the simulation feel more like scanning a real patient or aspects that made  • Aspects that made using the equipment unwieldy | ‘Could be enhanced with a TV scan to confirm diagnostic accuracy’ and ‘The scanner was helpful although shaky at times’. [P3]  ‘Improvement in the equipment will make better sense.’ [P5]  ‘Perhaps the patient could have more fat? Or larger to aid visualisation’ [P12]  ‘…as BodyWorks Eve is immobile and the technical controls are very different to an US machine. It was difficult to portray what I can do in clinical practice with BodyWorks Eve.’ [P13]  ‘It would be helpful if the programme did not automatically delete my annotations’ [P4]  ‘…found it more difficult to scan dummy so become more adapted to it’ [P8]  ‘Tendency to forget things in this scenario since the patient is not real and less adaptions.’ [P5] |
